# 3′5-Dimaleamylbenzoic Acid Attenuates Bleomycin-Induced Pulmonary Fibrosis in Mice

**DOI:** 10.3390/ijms23147943

**Published:** 2022-07-19

**Authors:** Karina González-García, Armando López-Martínez, Juan Manuel Velázquez-Enríquez, Cecilia Zertuche-Martínez, Gabriela Carrasco-Torres, Luis Manuel Sánchez-Navarro, Saúl Villa-Treviño, Rafael Baltiérrez-Hoyos, Verónica Rocío Vásquez-Garzón

**Affiliations:** 1 Laboratorio de Fibrosis y Cáncer, Facultad de Medicina y Cirugía, Universidad Autónoma “Benito Juárez” de Oaxaca, Ex Hacienda de Aguilera S/N, Sur, Oaxaca C.P. 68020, Mexico; k.igg@cecad-uabjo.mx (K.G.-G.); armandoloopez37@cecad-uabjo.mx (A.L.-M.); juanmanuelvela_enriquez@cecad-uabjo.mx (J.M.V.-E.); cecizertuche95@cecad-uabjo.mx (C.Z.-M.); 2 Departamento de Nanociencias y Nanotecnología, Centro de Investigación y de Estudios Avanzados del IPN, Av. IPN 2508, Ciudad de México C.P. 07360, Mexico; gabriela.carrasco@cinvestav.mx; 3 Facultad de Medicina y Cirugía, Universidad Autónoma “Benito Juárez” de Oaxaca, Ex Hacienda de Aguilera S/N, Sur, Oaxaca C.P. 68020, Mexico; sluismanuel81@hotmail.com; 4 Departamento de Biología Celular, Centro de Investigación y de Estudios Avanzados del Instituto Politécnico Nacional, Ciudad de Mexico C.P. 07360, Mexico; svilla@cell.cinvestav.mx; 5 CONACYT-Facultad de Medicina y Cirugía, Universidad Autónoma “Benito Juárez” de Oaxaca, Ex Hacienda de Aguilera S/N, Sur, Oaxaca C.P. 68020, Mexico

**Keywords:** myofibroblasts, α-SMA, collagen, transforming growth factor-β1, oxidative stress, pro-oxidants

## Abstract

Idiopathic pulmonary fibrosis (IPF) is a chronic lung disease characterized by parenchymal scarring, leading progressively to alveolar architecture distortion, respiratory failure, and eventually death. Currently, there is no effective treatment for IPF. Previously, 3′5-dimaleamylbenzoic acid (3′5-DMBA), a maleimide, demonstrated pro-apoptotic, anti-inflammatory, and anti-cancer properties; however, its potential therapeutic effects on IPF have not been addressed. Bleomycin (BLM) 100 U/kg was administered to CD1 mice through an osmotic minipump. After fourteen days of BLM administration, 3′5-DMBA (6 mg/kg or 10 mg/kg) and its vehicle carboxymethylcellulose (CMC) were administered intragastrically every two days until day 26. On day 28, all mice were euthanized. The 3′5-DMBA effect was assessed by histological and immunohistochemical staining, as well as by RT-qPCR. The redox status on lung tissue was evaluated by determining the glutathione content and the GSH/GSSG ratio. 3′5-DMBA treatment re-established typical lung histological features and decreased the expression of BLM-induced fibrotic markers: collagen, α-SMA, and TGF-β1. Furthermore, 3′5-DMBA significantly reduced the expression of genes involved in fibrogenesis. In addition, it decreased reduced glutathione and increased oxidized glutathione content without promoting oxidative damage to lipids, as evidenced by the decrease in the lipid peroxidation marker 4-HNE. Therefore, 3′5-DMBA may be a promising candidate for IPF treatment.

## 1. Introduction

Idiopathic pulmonary fibrosis (IPF) is a chronic, disabling interstitial lung disease of unknown etiology [1]. It is characterized by healthy lung parenchymal tissue replacement with fibrotic stroma, loss of normal architecture, progressive dyspnea, and respiratory failure that eventually leads to death [2]. It is a disease with a poor prognosis, which is even worse than that of several types of cancer, with a median survival of 2 to 5 years after diagnosis [3]. Currently, only two drugs, nintedanib, and pirfenidone, have been approved by the Food and Drug Administration (FDA); however, they only slow IPF progression, and although they positively improve patient quality of life, they are not observed to reduce their mortality rates significantly [4]. Thus, there is an urgent need to identify new potential therapeutic agents for IPF patients.

Although the pathogenic mechanisms are not fully understood, evidence suggests the coexistence of damage and repair of the injured alveolus and aberrant extracellular matrix (ECM) deposition secreted by myofibroblasts in the interstitial space [4,5]. Myofibroblasts are mesenchymal cells that, upon activation, acquire a prominent contractile phenotype, generate stress fibers that incorporate high concentrations of alpha-smooth muscle actin (α-SMA), and are highly collagen producing [6]. Growing evidence shows that transforming growth factor-beta 1 (TGF-β1) is the critical fibrogenic factor in IPF, increasing its expression, promoting alveolar epithelial cells apoptosis, and facilitating epithelial-mesenchymal transition (EMT), which contributes to myofibroblasts expansion from epithelial cells [7]; and its persistence in lung tissue is associated with chronic and progressive fibrosis [8].

Reactive oxygen species (ROS) is a general term for many molecular oxygen derivatives produced in aerobic organisms [9]. ROS participate in IPF by activating latent TGF-β1, EMT, and driving myofibroblast differentiation [10,11,12]. ROS accumulation induces cellular damage in the absence of regulatory mechanisms. ROS interact with biomolecules such as DNA, RNA, proteins, and lipids, and their by-products are used as markers of oxidative damage. In this regard, increased levels of 4-hydroxy-2-nonenal (4-HNE), a marker of oxidative lipid damage, have been observed in IPF lung sample [13,14]. Glutathione (GSH) is a hydrophilic tripeptide that acts as the most critical antioxidant and protects cells against endogenous and exogenous toxicants, such as ROS [15]. Its loss leads to toxic oxidative stress (OS) state that can be estimated by the ratio of the reduced/oxidized form of glutathione (GSH/GSSG ratio) [16].

Animal models represent an essential tool to study new candidates for therapeutic agents for IPF. Bleomycin (BLM), a glycopeptide antibiotic produced by Streptomyces verticillus, is used to treat different types of neoplasms. However, among its most serious side effects are pulmonary toxicity and pathological changes compatible with IPF due to the absence of BLM hydrolase in the lung, an enzyme responsible for inactivating BLM. In this regard, BLM is widely used as an agent to induce experimental pulmonary fibrosis [17]. Moreover, the BLM-induced IPF model is the most commonly used due to its ability to replicate the pulmonary histological pattern observed in patients treated with this antineoplastic, characterized by patchy lung parenchymal inflammation, alveolar epithelial cell damage, ROS generation, EMT activation, differentiation of fibroblasts to myofibroblasts, and subsequent fibrosis [18,19]. It reproduces the inflammatory, fibroproliferative, and pulmonary fibrotic stages in relatively short intervals. The established fibrosis phase occurs from day 14 to 28, so it is the window of opportunity to evaluate the therapeutic antifibrotic potential and mimic the clinical situation [20,21].

According to the critical fibrogenic mechanisms, the priority therapeutic targets are inhibiting lung fibroblast proliferation and activation, inducing apoptosis, or dedifferentiation of myofibroblasts [22]. Cells that acquire resistance to apoptosis usually alter their metabolic pathways to adapt to the environment they find themselves in; dysregulation of the redox state is a common feature of carcinogenic cells [23]. Likewise, myofibroblasts acquire resistance to tissue clearance mechanisms through different pathways [24]; notably, proteomic studies reveal up-regulation of proteins involved in antioxidant defense, such as glutathione peroxidases, superoxide dismutases, and components of the glutathione biosynthetic pathway, suggesting protection against OS-regulated apoptosis [25]. According to this premise, inhibition of components of the antioxidant system could increase OS in cells with dysregulated redox states as myofibroblasts and bring them to the limit of antioxidant resistance, favoring their sensitization to clearance.

Maleimides are potential therapeutic agents against cancer with high selectivity [26]. They are pro-oxidants by deactivating intracellular cysteines, particularly glutathione cysteines; they lead to increased OS, cytotoxicity, and cell apoptosis [26,27,28]. Previously, 3′5-dimaleamylbenzoic acid (3′5-DMBA), a maleimide, has been shown to inhibit proliferation and induce apoptosis of hepatocellular carcinoma cells by depleting glutathione and increasing OS without affecting non-tumor hepatocytes [29]; however, its potential impact on IPF has not been addressed.

In this work, we explore for the first time the pharmacological role of 3′5-DMBA in a BLM-induced pulmonary fibrosis model. Our results showed that 3′5-DMBA attenuates histopathological changes characteristic of IPF and modulates the expression of profibrotic mediators at the genetic and protein levels, attributing to 3′5-DMBA a possible antifibrotic potential.

## 2. Results

### 2.1. 3′5-DMBA Attenuates BLM-Induced Pulmonary Fibrosis in Mice

**Figure 1:**
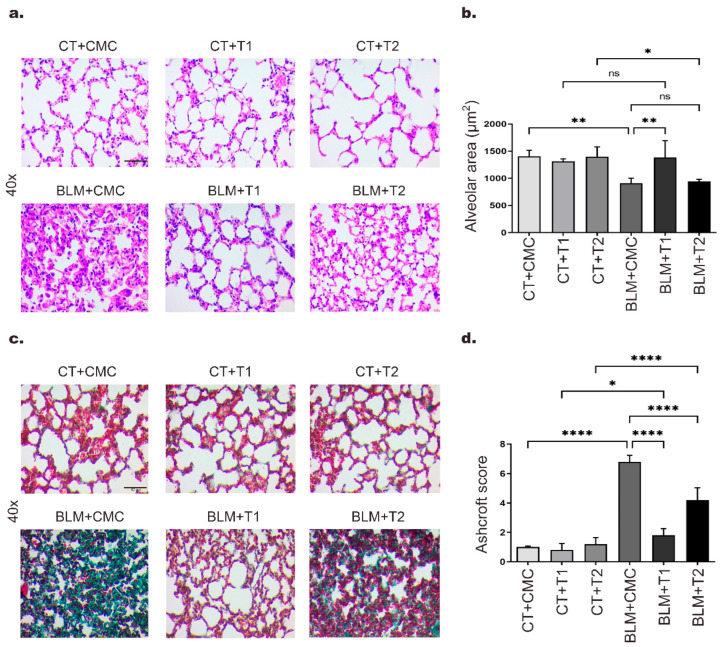
3′5-DMBA attenuates BLM-induced pulmonary fibrosis in mice. Mice in BLM groups were instilled with an osmotic minipump loaded with BLM 100 U/kg. Mice in control groups (CT) have a sham instillation. On day 14, mice received either 3′5-DMBA or its vehicle intragastrically until they were euthanized on day 28. (**a**) Sections of lung tissue stained with H&E; (**b**) bar graph showing H&E staining analysis of alveolar area size made using ImageJ software; (**c**) Masson’s trichrome staining of lung tissue showing collagen fibers in green; (**d**) Bar graph showing the Ashcroft scale score for all groups. CT+CMC: control group, BLM+CMC: BLM-treated group, BLM+T1: BLM plus 3′5-DMBA-treated group at a dose of 6 mg/kg, and BLM+T2: BLM plus 3′5-DMBA-treated group at a dose of 10 mg/kg. Representative images were taken at 40× magnification. Scale bar = 50 μm. Data are represented as the mean ± SD (*n* = 4). Data were analyzed by one-way ANOVA followed by Tukey’s *t*-test for comparison between groups. For each graph, the asterisks above the horizontal lines indicate significant differences with respect to their control groups and differences between the two doses and the vehicle. * *p* < 0.05, ** *p* < 0.01, and **** *p* < 0.0001. BLM: bleomycin, CMC: carboxymethylcellulose (vehicle), and 3′5-DMBA: 3′5-dimaleamylbenzoic acid.

To evaluate the effect of 3′5-DMBA on lung fibrosis, we used the BLM induction model. For this purpose, CD1 mice in the BLM groups were treated with BLM (100 U/kg) using an osmotic minipump, defining the day of BLM administration as day 0. On day 14 post-induction, the corresponding volume of 0.05% carboxymethyl cellulose (CMC) vehicle was administered intragastrically in the CT+CMC and BLM+CMC groups. In addition, 3′5-DMBA was administered intragastrically at 6 mg/kg (T1) or 10 mg/kg (T2) in the CT+T1 and BLM+T1 or CT+T2 and BLM+T2 groups, respectively. Treatments were administered every two days until sacrifice on day 28. Histological changes were assessed by H&E staining. The results showed that the CT+CMC control group presented a well-defined lung structure with standard alveolar septa and air spaces. The CT+T1 and CT+T2 groups showed no histological alterations (Figure 1a). Consequent to the BLM lesion, the BLM+CMC group presented loss of typical alveolar structure with alveolar wall thickening and alveolar area reduction (Figure 1a). Meanwhile, in the BLM+T1 group, BLM-induced alveolar structure alterations decreased after treatment with 3′5-DMBA (6 mg/kg), and for the BLM+T2 group, minor attenuation effects of BLM-induced lung damage were observed when using a dose of 10 mg/kg 3′5-DMBA (Figure 1a,b). Masson’s trichrome staining, which allows collagen fibers to be observed in green, was performed to evaluate collagen deposition. The lungs of the BLM+CMC group showed increased collagen-positive staining compared to control groups, while the BLM+T1 group showed decreased collagen deposition compared to the BLM+CMC group. The BLM+T2 group showed no significant decrease in collagen compared to the BLM+CMC group (Figure 1c). In addition, the BLM+CMC group presented an Ashcroft score of 6.8, compared to the score of 1 for the CT+CMC group. After 3′5-DMBA intervention, the BLM+T1 and BLM+T2 groups obtained an Ashcroft score of 1.8 and 4.2 compared to their control groups, CT+T1 and CT+T2 achieved a score of 0.8 and 1.2, respectively (Figure 1d). These results suggest that 3′5-DMBA decreases lung injury and attenuates BLM-induced lung fibrosis with better effects at the 6 mg/kg dose.

### 2.2. 3′5-DMBA Reduces Pulmonary Fibrosis-Related Protein Expression in Mice

**Figure 2:**
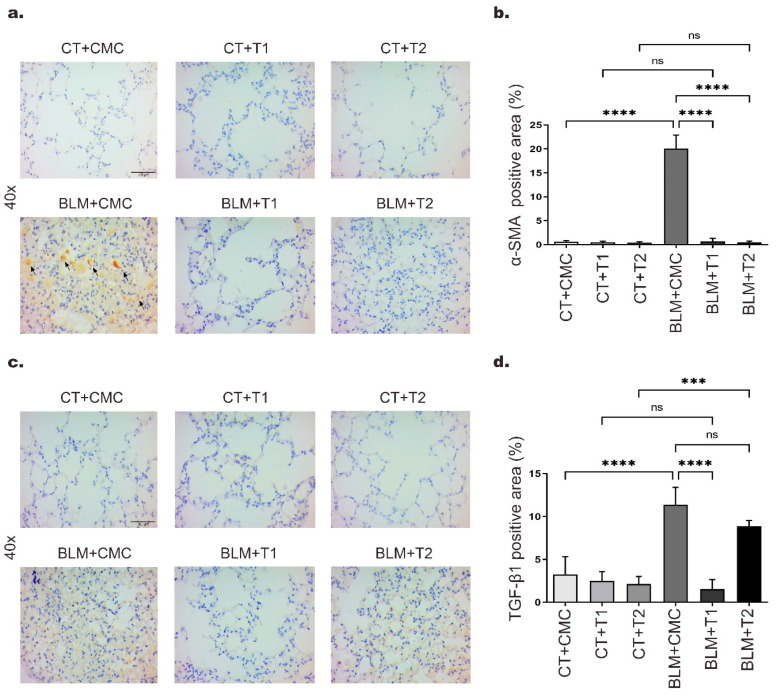
3′5-DMBA reduces proteins related to pulmonary fibrosis expression in mice. Mice in BLM groups were instilled with an osmotic minipump loaded with BLM 100 U/kg. Mice in control groups (CT) have a sham instillation. On day 14, mice received either 3′5-DMBA or its vehicle intragastrically until they were euthanized on day 28. (**a**,**c**) Immunohistochemical staining for α-SMA and TGF-β1 in lung tissue sections, respectively. Positive staining is shown in brown. Arrows show fibroblastic foci. (**b**,**d**) Bar graph showing immunohistochemical staining analysis for α-SMA and TGF-β1 represented as the percentage of positive area made using ImageJ software. CT+CMC: control group, BLM+CMC: BLM-treated group, BLM+T1: BLM plus 3′5-DMBA-treated group at a dose of 6 mg/kg, and BLM+T2: BLM plus 3′5-DMBA-treated group at a dose of 10 mg/kg. Representative images were taken at 40× magnification. Scale bar = 50 μm. Data are represented as the mean ± SD (*n* = 4). Data were analyzed by one-way ANOVA followed by Tukey’s *t*-test for comparison between groups. For each graph, the asterisks above the horizontal lines indicate significant differences with respect to their control groups and differences between the two doses and the vehicle. *** *p* < 0.001, and **** *p* < 0.0001. BLM: bleomycin, CMC: carboxymethylcellulose (vehicle), and 3′5-DMBA: 3′5-dimaleamylbenzoic acid.

Proliferation, activation, and differentiation of fibroblasts to myofibroblasts are early events in tissue repair and contribute to IPF development [2]. To evaluate the effect of 3′5-DMBA on lung fibrogenesis, we performed immunohistochemical analysis for α-SMA, a recognized marker of myofibroblast activation. In immunohistochemical assays, the positive area is observed in brown gradients. Our results showed that after BLM treatment, α-SMA expression increased up to 19.5-fold in the BLM+CMC group relative to the CT+CMC group, whereas it decreased 19.4 and 19.42-fold, respectively, in the BLM+T1 and BLM+T2 groups compared to the BLM+CMC group (Figure 2a,b). Critical events in fibrogenesis, such as myofibroblast activation, are primarily orchestrated by TGF-β1, the major profibrotic cytokine [12]. Immunohistochemical staining for TGF-β1 showed that in the BLM+CMC group, its expression was increased up to 7.6-fold compared to the CT+CMC group. In contrast, the BLM+T1 group showed a 9.3-fold decrease in TGF-β1 expression relative to the BLM+CMC group (Figure 2c,d). The attenuation effect of TGF-β1 in the BLM+T2 group was 3.3-fold that in the BLM+CMC group. These results suggest that treatment with 3′5-DMBA can decrease the expression of pulmonary fibrosis-related proteins in a concentration-dependent manner, thus exerting an antifibrotic effect.

### 2.3. 3′5-DMBA Modulates Gene Expression Related to BLM-Induced Pulmonary Fibrosis in Mice

**Figure 3:**
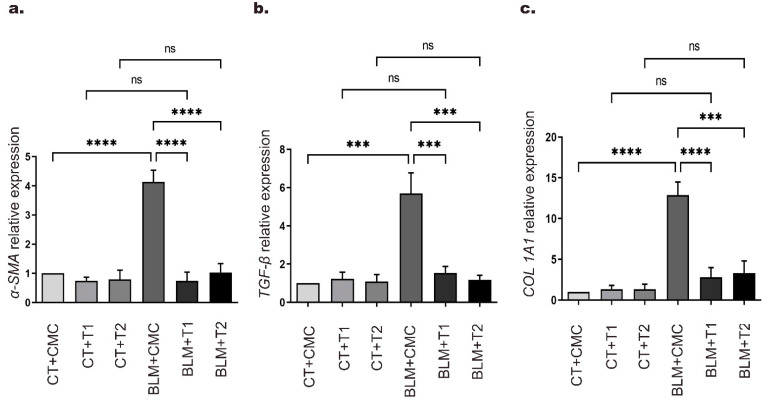
3′5-DMBA modulates gene expression related to BLM-induced lung fibrosis in mice. Mice in BLM groups were instilled with an osmotic minipump loaded with BLM 100 U/kg. Mice in control groups (CT) have a sham instillation. On day 14, mice received either 3′5-DMBA or its vehicle intragastrically until they were euthanized on day 28. The mRNA levels of (**a**) *α-SMA*; (**b**) *TGF-β1*, and (**c**) *COL1A1*, were determined by real-time quantitative PCR (RT-qPCR) in mouse lung tissue. CT+CMC: control group, BLM+CMC: BLM-treated group, BLM+T1: BLM plus 3′5-DMBA-treated group at a dose of 6 mg/kg, and BLM+T2: BLM plus 3′5-DMBA-treated group at a dose of 10 mg/kg. Data are represented as the mean ± SD (*n* = 4). Data were analyzed by one-way ANOVA followed by Tukey’s *t*-test for comparison between groups. For each graph, the asterisks above the horizontal lines indicate significant differences with respect to their control groups and differences between the two doses and the vehicle.*** *p* < 0.001, and **** *p* < 0.0001. BLM: bleomycin, CMC: carboxymethylcellulose (vehicle), and 3′5-DMBA: 3′5-dimaleamylbenzoic acid.

Our previous results in histological and immunohistochemical staining showed that 3′5-DMBA reduces the expression of fibrosis-related proteins: α-SMA, TGF-β1, and collagen. To further investigate the mechanisms by which 3′5-DMBA may regulate these proteins, we evaluated the expression of their respective genes by RT-qPCR. Our results showed that BLM administration in the BLM+CMC group increased *α-SMA* gene expression 3.4-fold compared to the CT+CMC group. On the other hand, *TGF-β* gene expression increased 4.7-fold in the BLM+CMC group relative to the CT+CMC group. Finally, collagen 1A1 (*COL1A1*) gene expression increased 11.9-fold in the BLM+CMC group compared to the CT+CMC group. For all three cases, treatment with both doses of 3′5-DMBA significantly reversed the expression of these genes to similar values of their respective control groups (Figure 3a–c). Therefore, these results suggest that 3′5-DMBA regulates the expression of profibrotic genes as part of its beneficial effects on pulmonary fibrosis.

### 2.4. 3′5-DMBA Modulates the Expression of Genes Related to Cell Proliferation and Remodeling of the ECM during Its Antifibrotic Effect

**Figure 4:**
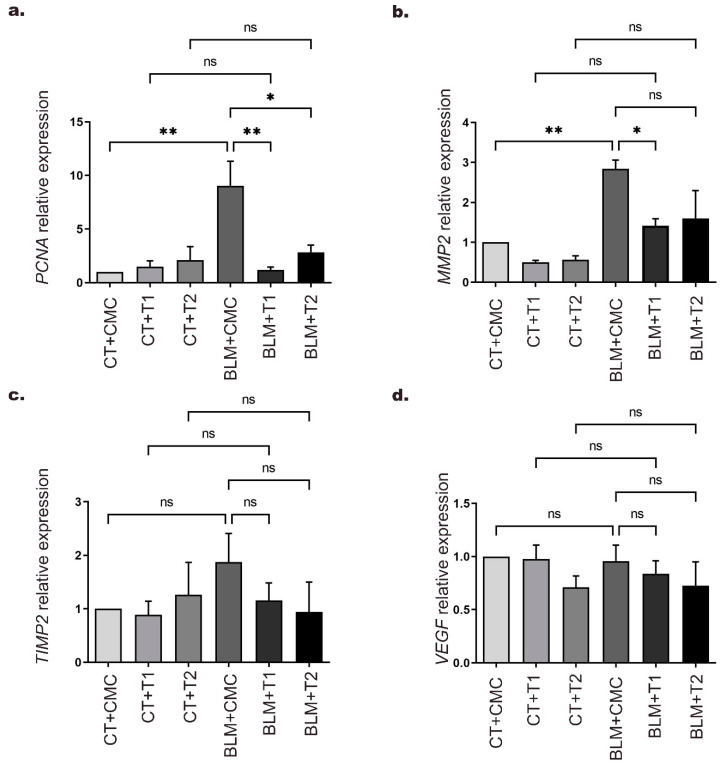
3′5-DMBA modulates the expression of genes related to cell proliferation and remodeling of the ECM during its antifibrotic effect. Mice in BLM groups were instilled with an osmotic minipump loaded with BLM 100 U/kg. Mice in control groups (CT) have a sham instillation. On day 14, mice received either 3′5-DMBA or its vehicle intragastrically until they were euthanized on day 28. The mRNA levels of (**a**) *PCNA*; (**b**) *MMP2*; (**c**) *TIMP2*; and (**d**) *VEGF*, were determined in mouse lung tissue by real-time quantitative PCR (RT-qPCR). CT+CMC: control group, BLM+CMC: BLM-treated group, BLM+T1: BLM plus 3′5-DMBA-treated group at a dose of 6 mg/kg, and BLM+T2: BLM plus 3′5-DMBA-treated group at a dose of 10 mg/kg. Data are represented as the mean ± SD (*n* = 4). Data were analyzed by one-way ANOVA followed by Tukey’s *t*-test for comparison between groups. For each graph, the asterisks above the horizontal lines indicate significant differences with respect to their control groups and differences between the two doses and the vehicle. * *p* < 0.05, ** *p* < 0.01. BLM: bleomycin, CMC: carboxymethylcellulose (vehicle), and 3′5-DMBA: 3′5-dimaleamylbenzoic acid.

Aberrant fibroblast proliferation is a feature of IPF development [1]. Therefore, in order to evaluate cell proliferation and the effect of 3′5-DMBA on this central event, we measured *PCNA* gene expression by RT-qPCR. Our results showed that *PCNA* gene expression was increased up to 8-fold in the BLM+CMC group relative to the CT+CMC group. At the same time, the BLM+T1 and BLM+T2 groups exhibited a 7.8 and 6.1-fold reduction, respectively, after treatment with 3′5-DMBA compared to the BLM+CMC group (Figure 4a).

Moreover, myofibroblast accumulation in the interstitial space drives ECM accumulation mediated by dysregulation between matrix metalloproteinases (MMPs) and their inhibitors (TIMPs) [30]. Therefore, to evaluate the effect of 3′5-DMBA on MMPs and TIMPs, we assessed the expression of *MMP2* and *TIMP2* genes reported to be related to this process. Our results showed that *MMP2* expression in the BLM+CMC group increased up to 1.8-fold compared to the CT+CMC group, whereas this expression was reduced 1.4-fold in the BLM+T1 group after 3′5-DMBA treatment relative to the BLM+CMC group. However, the BLM+T2 group did not show significant changes in *MMP2* expression as compared to the BLM+CMC group (Figure 4b). For the *TIMP2* gene, no significant differences were observed in any of the BLM-treated groups relative to their control groups (Figure 4c).

Finally, angiogenesis is an event that occurs during fibrogenesis and is mainly assessed by vascular endothelial growth factor (VEGF) expression [31]. Therefore, in order to explore the effect of 3′5-DMBA on VEGF, we evaluated its gene expression by RT-qPCR. Our results showed no significant changes in any experimental group (Figure 4d). Taken together, these results suggest that 3′5-DMBA modulates the expression of *PCNA* and *MMP2* genes that drive cell proliferation and ECM accumulation, respectively, as part of its attenuating effect in IPF.

### 2.5. 3′5-DMBA Exerts Pro-Oxidant Effects without Promoting Increased Lipid Peroxidation

**Figure 5:**
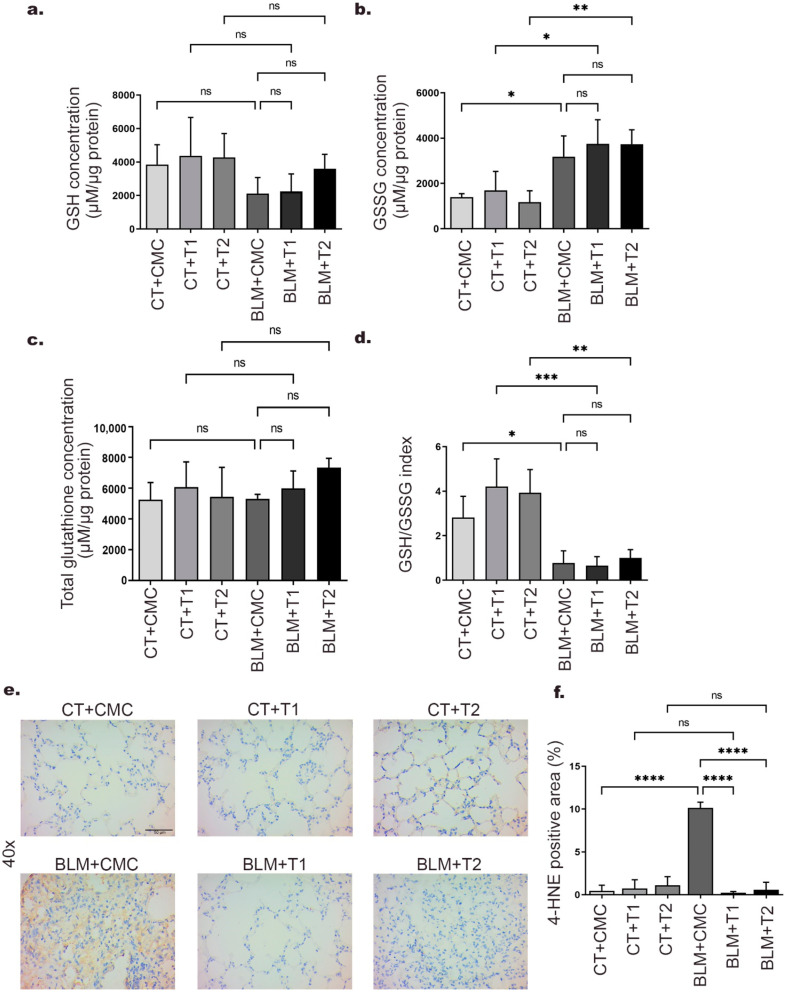
3′5-DMBA can exert pro-oxidant effects without favoring increased lipid peroxidation. Mice in BLM groups were instilled with an osmotic minipump loaded with BLM 100 U/kg. Mice in control groups (CT) have a sham instillation. On day 14, mice received either 3′5-DMBA or its vehicle intragastrically until they were euthanized on day 28. Oxidative stress parameters, including (**a**) GSH concentration, (**b**) GSSG concentration, (**c**) total glutathione concentration, and (**d**) GSH/GSSG ratios, were determined from lung tissue by colorimetric assays. (**e**) Immunohistochemical staining for 4-HNE in lung tissue sections. Positive staining is shown in brown. (**f**) Bar graph showing immunohistochemical staining analysis for 4-HNE represented as the percentage of positive area made using ImageJ software. CT+CMC: control group, BLM+CMC: BLM-treated group, BLM+T1: BLM plus 3′5-DMBA-treated group at a dose of 6 mg/kg, and BLM+T2: BLM plus 3′5-DMBA-treated group at a dose of 10 mg/kg. Representative images were taken at 40× magnification. Scale bar = 50 μm. Data are represented as the mean ± SD (*n* = 4). Data were analyzed by one-way ANOVA followed by Tukey’s *t*-test for comparison between groups. For each graph, the asterisks above the horizontal lines indicate significant differences with respect to their control groups and differences between the two doses and the vehicle. * *p* < 0.05, ** *p* < 0.01, *** *p* < 0.001, and **** *p* < 0.0001. BLM: bleomycin, CMC: carboxymethylcellulose (vehicle), and 3′5-DMBA: 3′5-dimaleamylbenzoic acid.

Pro-oxidants are molecules that decrease antioxidant activity and availability or increase ROS generation [32]. Previously, 3′5-DMBA has been reported to reduce glutathione availability in tumor cells [29]. Therefore, to evaluate the effect of 3′5-DMBA on tissue glutathione content in our model, we determined the reduced, oxidized, and total glutathione concentration by colorimetric assays. On the one hand, our results show that GSH concentration decreased 45.1%, 48.6%, and 15.7% in the BLM+CMC, BLM+T1, and BLM+T2 groups, respectively compared to their control groups CT+CMC, CT+T2, and CT+T2 (Figure 5a). On the other hand, an increase in GSSG concentration of 56.1%, 54.9%, and 68.6% was observed in the BLM+CMC, BLM+T1, and BLM+T2 groups, respectively, compared to their control groups CT+CMC, CT+T2, and CT+T2 (Figure 5b). For total glutathione level, no significant changes were observed in any of the BLM+CMC, BLM+T1, and BLM+T2 groups compared to their control groups (Figure 5c). With these results, we calculated the GSH/GSSG ratio, an oxidative status indicator. Our results show a 72.5%, 84.5%, and 74.5% reduction in the GSH/GSSG ratio of the BLM+CMC, BLM+T1, and BLM+T2 groups, respectively, compared to their control groups CT+CMC, CT+T1, and CT+T2 (Figure 5d).

In addition, lipid peroxidation (LPO) is an OS consequence and is causally associated with the development of IPF [14]. Our previous results show that 3′5-DMBA does not decrease BLM-induced OS. Therefore, in order to evaluate the effect of 3′5-DMBA on lipid damage, immunohistochemical staining for 4-HNE as a by-product of LPO was performed. Our results show that after BLM treatment, 4-HNE expression increased 9.7-fold in the BLM+CMC group relative to their control CT+CMC group. After 3′5-DMBA treatment, 4-HNE expression in the BLM+T1 and BLM+T2 groups decreased 9.9 and 9.5-fold compared to the BLM+CMC group (Figure 5e,f). Therefore, taken together, these results suggest that although 3′5-DMBA cannot rescue from BLM-induced OS, it does not promote LPO increase.

## 3. Discussion

IPF is a chronic, disabling, progressive disease with a poor prognosis that remains without effective therapies [2]. Although fibrogenesis knowledge is still limited, it is recognized that it involves fibroblast activation, increased myofibroblast number and activity, secretion of profibrotic factors, the activity of remodeling enzymes, and ROS that, in an exacerbated manner, promote alveolar architecture loss and lung activity impairment [5]. The BLM-induced IPF model is the most widely used to evaluate different phases of fibrogenesis and the potential therapeutic effect of compounds in each of them [19]. In the present study, we evaluated the possible therapeutic effect of 3′5-DMBA in a BLM-induced pulmonary fibrosis model. As characteristics of this model, acute lung injury with increased production of proinflammatory cytokines and inflammatory infiltrate is observed from day 0 to 7; a fibroproliferative phase from day 3 to 14; established fibrosis from day 14 to 28, and spontaneous resolution at variable periods [21,33]. According to the American Thoracic Society (ATS) consensus, the effect of an antifibrotic should be evaluated after the acute inflammatory phase in response to BLM stimulation when there is histologic evidence of fibrosis [34]. Concordantly, intragastric treatment with 3′5-DMBA started on day 14 after BLM treatment, when the fibrotic phase had begun. Our results suggest that 3′5-DMBA attenuates histopathological changes in lung tissue, mainly by regulating the expression of fibrogenic factors at both the genetic and protein levels. Histological staining showed that the typical distortion of alveolar architecture and deposition of high collagen concentrations promoted by BLM were attenuated by 3′5-DMBA treatment mainly at the dose of 6 mg/kg; therefore, our results suggest that 3′5-DMBA has potential antifibrotic effects in a pulmonary fibrosis model. Maleimides exhibit anti-inflammatory, antiproliferative, and proapoptotic effects that are related to their anticarcinogenic potential [26]. 3′5-DMBA has shown strong cytotoxic capacity on HuH7 and HepG2 hepatocarcinoma cells, while no detrimental effects were observed on non-tumor liver cells [29,35]. These results suggest the selectivity of the effect exerted and indicate the possibility of a treatment approach that has no harmful effects on normal cells. Our results show no histological changes in the lungs of the control groups treated with 3′5-DMBA (CT+T1 and CT+T2), suggesting that it exerts no toxic effects. Consistent with our results, the study by Aastha Arora et al. demonstrated that DRDE-30 exerts a beneficial impact on the resolution of BLM-induced pulmonary fibrosis without altering the histology of healthy lungs [36]. Similarly, isorhamnetin alleviated experimental IPF with an acceptable safety profile [37].

Myofibroblasts are critical cells in IPF that secrete fibrogenic factors, remodeling enzymes, and collagen [6]; they are usually grouped parallel to the alveolar surface in fibroblastic foci [38]. Increased fibroblastic foci are associated with poor prognosis and elevated mortality [39]. Therefore, a therapeutic approach that decreases myofibroblast viability and activity could result in effective therapy [4]. Upon activation, myofibroblasts acquire a highly contractile phenotype characterized by α-SMA overexpression, the loss of which is one of the initial processes for fibrosis resolution [40]. However, IPF resolution is complex due to myofibroblast apoptosis resistance. Our results show that administration of both doses of 3′5-DMBA downregulates *α-SMA* gene expression and consistently the number of α-SMA-positive fibroblastic foci in lung tissue. Clearance of myofibroblasts by dedifferentiation and restoration of sensitivity to apoptosis proceeds under different transcriptomic transitions [22]. The loss of α-SMA expression and the number of fibroblastic foci is in line with decreased collagen fiber deposition, suggesting that 3′5-DMBA exerts beneficial effects for IPF resolution.

Maleimides exhibit anti-inflammatory, antiproliferative, and proapoptotic effects related to their anticarcinogenic potential [26]. Treatment of hepatocarcinoma cells with 3′5-DMBA favors cell cycle arrest in the G0/G1 phase and expression of apoptotic caspases involved with intrinsic pathway activation; caspases 9 and 3, following increased levels of ROS and OS [29]; therefore, the imbalance between antioxidants and pro-oxidants results in detrimental effects culminating in apoptosis. Dysregulation of antioxidant systems in the population with myofibroblastic phenotype could make them an eligible target for the effect of 3′5-DMBA. Therefore, myofibroblast clearance by apoptosis may be one of the effects of 3′5-DMB treatment, consistent with the decrease in α-SMA-positive cells in lung tissue.

On the other hand, perturbation of the antioxidant balance may lead to adaptive responses depending on the severity of the damage caused by increased ROS [41]. It is now recognized that myofibroblasts can dedifferentiate under the stimulus of prostaglandin E and fibroblast growth factor-2. Although both molecules drive dedifferentiation, it usually occurs under different transcriptional programs [22]. According to the basic criteria for dedifferentiation; loss of α-SMA, and collagen expression, our results show that 3′5-DMBA decreases its gene and protein expression during its pharmacological effect, so it could promote myofibroblast dedifferentiation. Although these findings are promising, it is important to accept that they are assumptions that need to be confirmed experimentally.

On the other hand, myofibroblast differentiation and activity occur mainly under the TGF-β1 stimulus, which modulates EMT, collagen synthesis, and α-SMA expression [8]. TGF-β overexpression in murine models is the most potent stimulus for myofibroblast differentiation and thus IPF development. Its ablation allows alveolar restructuring and downregulation of fibrogenic factors associated with its signaling [7]. Decreased TGF-β expression in lung tissue prevents fibroblast differentiation, favors inhibition of profibrotic functions, and reduced tissue collagen deposition [42]. Accordingly, our findings demonstrate that BLM treatment increases TGF-β gene and protein expression, whereas 3′5-DMBA treatment attenuates its expression. Therefore, we can speculate that this decrease prevents fibroblast differentiation as part of its beneficial effect on IPF.

ECM remodeling involves regulation of MMP activity precisely at the transcriptional level, zymogen activation, and inhibition by TIMPs; however, during IPF, it is deregulated [30]. Increased MMP2 following BLM damage has been reported previously as a p53 mechanism of action in response to DNA damage [43]. Consistently, our results showed increased *MMP2* gene expression in the BLM+CMC group. While both doses of 3′5-DMBA downregulate *MMP2* gene expression, no effect was observed on its corresponding *TIMP2*; therefore, this decrease could be due to the alleviation of lung damage and alveolar restructuring brought about by 3′5-DMBA.

Pathological fibroblast proliferation is an IPF hallmark [1]. Therefore, we evaluated the expression of PCNA, a protein expressed during DNA replication in cell cycle progression and is widely used as a proliferation marker [44]. Our results showed that due to the BLM effect, the BLM+CMC group significantly increased *PCNA* expression compared to the control group. Moreover, 3′5-DMBA downregulated *PCNA* gene expression in the BLM+T1 and BLM+T2 groups relative to the BLM+CMC group. *PCNA* downregulation implies decreased cell proliferation in lung tissue. Based on these results, it is possible to propose that 3′5-DMBA decreases exacerbated fibroblast proliferation in line with our previous results on α-SMA, TGF-β, and collagen; however, more studies are needed to confirm this.

BLM toxicity is expressed as oxidative lung damage by overproduction of ROS and is strongly linked to antioxidant depletion. Consequently, OS is associated with IPF at the cellular, tissue, and systemic level [45]. Regulatory mechanisms that counteract this toxicity are responsible for maintaining cell survival and tissue function. GSH is a tripeptide with thiol groups on its reactive cysteines and acts as a potent free radical scavenger. After capturing these radicals, it is oxidized to their GSSG form [15]. Due to the pro-oxidant character of 3′5-DMBA, we evaluated its effect on GSH, GSSG, and total glutathione concentration in lung tissue homogenate. We observed that GSH concentration in the BLM+CMC group decreased compared to the CT+CMC group, as previously reported in the BLM model [46]. GSH decrease in BLM+T1 and BLM+T2 groups was less marked compared to the BLM+CMC group. GSSG concentration was significantly increased in the BLM+CMC, BLM+T1, and BLM+T2 groups compared to their controls. Meanwhile, total concentration remained without significant changes for all groups. The decrease in the GSH/GSSG ratio in these same groups suggests the existence of OS [47]. Considering that CT+T1 and CT+T2 groups did not present modifications in the concentration of the glutathione fractions, we proposed that the 3′5-DMBA effect is specifically on some cell types of the total homogenate. Therefore, we can hypothesize that the selectivity of 3′5-DMBA may be possible considering the high ROS and low GSH levels in specific cells of the lung homogenate; and the pro-oxidant character of 3′5-DMBA. In addition, elevated GSH levels in different cell lineages are associated with their apoptosis-resistant phenotype, similar to myofibroblasts in IPF. Intracellular GSH loss is one of the central processes in the progression toward cell death induced under different pro-apoptotic stimuli [48].

OS promotes structural and functional damage to DNA, proteins, and lipids. LPO actively participates in OS-mediated cellular damage and is represented by the overexpression of its by-products, particularly 4-HNE [13]. Our results of the 4-HNE immunohistochemical analysis show a significant increase in the BLM+CMC group compared to the CT+CMC group, consistent with other studies performed in murine models and samples from patients with lung diseases [49,50]. Surprisingly, BLM+T1 and BLM+T2 groups decreased 4-HNE expression compared to the BLM+CMC group, indicating that 3′5-DMBA does not increase oxidative damage to lipids despite its pro-oxidant character. Previous reports show an interrelationship between TGF-β and 4-HNE expression [51], so 3′5-DMBA could modulate 4-HNE expression by regulating TGF-β expression.

Our results showed better effects of 3′5-DMBA on pulmonary fibrosis at a low dose of 6 mg/kg compared to the high dose of 10 mg/kg. Previously, concentration-dependent variation in therapeutic effects has been reported as a response to stimulation at low doses and inhibition at high doses in compensation for mild stress; a phenomenon termed hormesis [52]. For example, the flavonoid quercetin has been shown to exert dual effects depending on the dose administered. Vásquez-Garzón et al. showed its potent chemoprotective effect on experimental hepatocarcinogenesis at doses below 10 mg/kg and inhibition at high concentrations of 25 and 50 mg/kg [53]. Nitrated fatty acid CXA-10 exhibits anti-inflammatory, antifibrotic, and renal injury-limiting effects with a hormetic dose–response profile [54]. Similarly, binaphthyl phosphonothioates, compounds with a pro-oxidant character, exhibit this hormetic behavior [52]. The beneficial effect of low doses of antifibrotic has been demonstrated in clinical trials with pirfenidone; in this regard, decreasing the dose of pirfenidone shows an improvement in forced vital capacity and survival of patients compared to the standard dose [55]. According to our findings, the effect of 3′5-DMBA may be possible at doses lower than 10 mg/kg. This phenomenon is not necessarily related to toxicity; consistently, we show a loss of attenuating effect on pulmonary fibrosis without increased tissue damage. Although the specific hormetic mechanisms are so far unclear, it is feasible that there is a post-translational regulation with activation of stress-susceptible targets or inactivation of less susceptible ones [54]. Therefore, our results highlight the need to investigate the dose–response relationship in future studies. The precise mechanisms mediating the antifibrotic effect of 3′5-DMBA need to be evaluated.

## 4. Materials and Methods

### 4.1. Animals

A total of 24 male CD1 mice, 8–10-weeks-old, and with 34–42 g body weight were obtained from the Laboratory Animal Production and Experimentation Unit (UPEAL) of CINVESTAV. All mice were acclimatized for 2 weeks and maintained in a pathogen-free environment with 12/12-h light/dark cycles. Standard food and beverages access ad libitum.

### 4.2. Experimental Design

**Figure 6:**
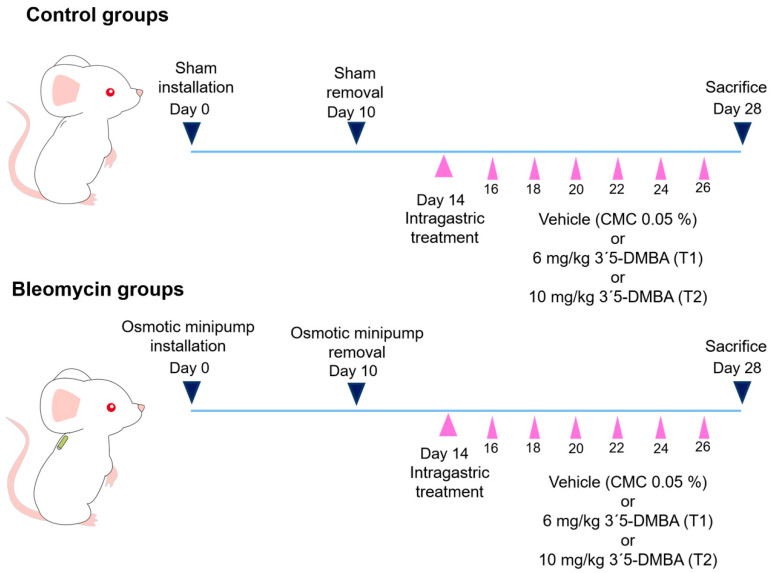
Schematic representation of experimental pulmonary fibrosis mice model. Subcutaneous instillation of the osmotic minipump loaded with bleomycin (BLM) 100 U/kg or a sham instillation was performed at day 0. On day 14, intragastric treatments were started every two days, as indicated by the purple arrows. On day 28, the mice of all experimental groups were sacrificed. CMC: carboxymethylcellulose (vehicle), and 3′5-DMBA: 3′5-dimaleamylbenzoic acid.

Mice were randomly divided into six groups with four mice each: three control groups (CT) and three bleomycin groups (BLM). Mice in the BLM groups were maintained under isofluorane anesthesia and instilled with an osmotic minipump (ALZET Durect, 1007D, Cupertino, CA, USA) loaded with BLM 100 U/kg (BLOMINDEX, Pisa, Guadalajara, Mexico) subcutaneously in the scapular area as previously reported [56]. Defining the day of BLM administration as day 0. Mice in the CT groups had sham instillation. On day 10, the osmotic minipump was removed from mice in BLM groups, while the control groups had a sham removal. On day 14 post-instillation, 3′5-DMBA at 6 mg/kg (T1) or 10 mg/kg (T2) was intragastrically administered in CT+T1 and BLM+T1 or CT+T2 and BLM+T2, respectively. The corresponding volume of carboxymethyl cellulose 0.05% (CMC) vehicle was administered intragastrically in the CT+CMC and BLM+CMC groups. Intragastric treatments were administered every two days until day 26. On day 28, all mice were euthanized under anesthesia (Figure 6).

### 4.3. Tissue Preparation

The left lobe was immediately cryopreserved with 2-methylbutane at −80 °C for subsequent analysis. The right lobe was used for histological and immunohistochemical studies. For this purpose, after fixation in 4% formalin, the tissue was dehydrated in increasing solutions of serial alcohols, followed by ethanol-xylene solution (1:1 ratio). The tissue was then embedded in paraffin and sectioned at 5 µm using a microtome (LEICA, RM2125RTS, Wetzlar, Germany), and the sections were collected on gelatinized slides for histological analysis. For immunohistochemical analysis, 3 µm lung sections were collected on silanized slides.

### 4.4. Histological Analysis

For H&E staining, slides were deparaffinized by heating for 30 min at 54 °C, then immersed in preheated xylene for 30 min. The tissues were then hydrated in decreasing concentrations of ethanol solutions and finally tap water. Slides were incubated in Harrys’ hematoxylin (HYCEL, 738, Guadalajara, Mexico) for 10 min, washed, and immersed for 1 s twice in an acid alcohol solution, followed by ammoniacal solution for 30 s, 96% ethanol for 5 min, and yellowish eosin (HYCEL, 688, Guadalajara, Mexico) for 10 min. Finally, the sections were incubated in 96% ethanol and mounted for microscopic analysis. For Masson Trichrome staining, we used the Masson-Goldner Trichrome kit (Merck Millipore, 100485, Burlington, MA, USA) according to the manufacturer’s instructions with some modifications. Briefly, after deparaffination tissues were incubated in Weigert’s hematoxylin solution (1:1 ratio) for 20 min and carefully washed with distilled water. Then, incubated with 1% acetic acid (Sigma Aldrich, A6283, St. Louis, MO, USA) for 5 s, incubated with reagent 1 for 10 min, washed with 1% acetic acid, reagent 2 for 1 min, washed with 1% acetic acid, reagent 3 for 30 min, quickly dipped in 96% alcohol twice, 1% acetic acid for 5 s, incubated in xylene for 5 min. Finally, the sections were dehydrated and mounted for further analysis. The severity of fibrosis was assessed using the previously reported Ashcroft score [57]. Briefly, images obtained from light microscopy at 40× magnification were scored from 0 (normal lung) to 8 (total fibrous field obliteration) with a total of 20 fields per slide. Finally, the scores of 4 slides per group were averaged.

### 4.5. Immunohistochemical Analysis

α-SMA, TGF-β1, and 4-HNE expression were determined by immunohistochemical analysis. Briefly, antigen retrieval was performed with 10 mM citrate buffer, pH 6, in a pressure cooker for 30 min. Subsequently, tissue sections were permeabilized by incubation in phosphate buffer saline (PBS) with 1% triton and washed with PBS. Endogenous peroxidase activity was inactivated with 6% H_2_O_2_ in methanol. Non-specific sites were blocked with 3% bovine serum albumin (BSA) in PBS for α-SMA, whereas for TGF-β1 and 4-HNE, it was 5%. In addition, primary antibodies were incubated as follows: rabbit polyclonal anti-α-SMA (1:200, Proteintech, 55135-1-AP, Rosemont, IL, USA), rabbit polyclonal anti-TGF-β1 (1:200, Abcam, ab92486, Cambridge, UK), and rabbit polyclonal anti-4-HNE (1:100, Abcam, ab46545, Cambridge, UK), overnight at 4 °C. Tissues were washed with PBS and incubated with rabbit anti-HRP secondary antibody (1:200, Invitrogen, 65-6120, Waltham, MA, USA) for 1 h at 37 °C. The signal was detected with the diaminobenzidine (DAB)-plus kit (Life Technologies, 00-2020, Carlsbad, CA, USA), then the sections were counterstained with Harris hematoxylin (HYCEL, 738, Guadalajara, Mexico), was dehydrated, and mounted with a mounting medium. The slides were observed under a light microscope at 40× magnification.

### 4.6. RT-qPCR

Total RNA was isolated from the left lung lobe using Quick-RNA Miniprep Plus Kit (Zymo Research, R1054, Los Angeles, CA, USA). RNA quantity and quality were measured using NanoDrop (Thermo Scientific, ND2000, Waltham, MA, USA). A260/A280 and A260/A230 ratios greater than 1.8, and 2, respectively, were considered to be of acceptable purity. According to the manufacturer’s instructions, RNA samples were subjected to reverse transcription using the ReverAid First Strand kit (Thermo Scientific, K1622, Waltham, MA, USA). Briefly, 2 μg of RNA was used in a total volume of 20 μL. The reaction was cycled 40 times at 25 °C for 10 min, 42 °C for 60 min followed by 70 °C for 5 min and finally by 4 °C for 5 min. RT-qPCR was performed in 40 thermal cycles in the following order: 15 s at 95 °C, 60 °C for 30 s, and 72 °C for 30 s in the StepOne (Applied Biosystems, 4376600, Waltham, MA, USA) using the SYBRgreen PCR master mix kit (Thermo Scientific, K0172, Waltham, MA, USA) in a total volume of 20 μL containing SYBRGreen master mix (2×) (Thermo Scientific, K0172, Waltham, MA, USA), primers, cDNA and nuclease-free water, according to the manufacturer’s protocol. Primers were designed with the CDTA oligo quest program and analyzed with VECTOR NTI advance 11.5.3 software. The presence of a single PCR product was verified by a single melting temperature peak and by detecting a band of the expected size on a 2% agarose gel. The sequences of the primer sets are as follows:

β-actin: Forward 5′-CAGCCTTCCTTCTTGGGTATG-3′ and Reverse 5′-GGCATAGAGGTCTTTACGGATG-3′. α-SMA: Forward 5′-TCAGGGAGTAATGGTTGGAATG-3′ and Reverse 5′-GGTGATGATGCCGTGTTCTA-3′. TGF-β: Forward 5′-CCTGAGTGGCTGTCTTTTGA-3′ and Reverse 5′-CGTGGAGTTTGTTATCTTTGCTG-3′. COLLAGEN 1A1: Forward 5′-CATAAAGGGTCATCGTGGCT-3′ and Reverse 5′-TTGAGTCCGTCTTTGCCAG-3′. MMP2: Forward 5′-GTTCAACGGTCGGGAATACA-3′ and Reverse 5′-GCCATACTTGCCATCCTTCT-3′. TIMP2: Forward 5′-CAGGAAAGGCAGAAGGAGATG-3′ and Reverse 5′-GATCATGGGACAGCGAGTG-3′. VEGF: Forward 5′-ATCTTCAAGCCGTCCTGTG-3′ and Reverse 5′-TCTCCTATGTGCTGGCTTTG-3′. PCNA: Forward 5′-CGAAGCACCAAATCAAGAGAAAG-3′ and Reverse 5′-CACCCGACGGCATCTTTATTA-3′. Relative quantification of gene expression was measured relative to the endogenous β-actin reference gene using the comparative 2-ΔΔCT method.

### 4.7. Total, Reduced (GSH), and Oxidized (GSSG) Glutathione Quantification

A homogenate of the left lung lobe was carried out for colorimetric assays. First, we performed the quantification of total protein in the homogenate using the Pierce BCA Protein Assay Kit (Thermo scientific, 23225, Ciudad de México, Mexico), according to the manufacturer’s instructions. Then, to determine the total, reduced, and oxidized glutathione concentrations, we conducted a colorimetric assay according to the manufacturer’s instructions (G-biosciences, 786-076, St. Louis, MO, USA). Briefly, centrifugation and recovery of the supernatant were performed after homogenizing the tissue in the sample buffer for total glutathione quantification. In a 96-well plate, 50 µL of each sample, 100 µL of working reagent were added in duplicate, incubated at room temperature, and then Ellman’s reagent was added. The plate was incubated in the dark for 25 min, and the signal was read at 420 nm. For oxidized glutathione (GSSG) measurements, samples were pretreated with 4-vinylpyridine to remove GSH in the sample (G-Biosciences, 786-031, St. Louis, MO, USA), and the exact steps were performed. The concentration of reduced glutathione was obtained by theoretical calculations, subtracting the concentration of oxidized glutathione from the concentration of total glutathione as indicated by the formula: GSH concentration = total glutathione-GSSG. The GSH/GSSG ratio was calculated using the data obtained from these measurements. Concentrations glutathione fractions are reported in relation to the total protein concentration in the homogenate, expressed as µM/μg.

### 4.8. Statistical Analysis

Histological sections were quantified with ImageJ 1.51j software. All results are expressed as the mean ± standard deviation. Statistical significance was assessed by one-way analysis of variance (ANOVA) followed by Tukey’s test performed with Prism v.7.0 software.; *p* < 0.05 was considered statistically significant.

## 5. Conclusions

Overall, 3′5-DMBA attenuates histopathological damage, myofibroblast marker expression, gene and protein expression of profibrotic mediators without increasing oxidative damage to lipids despite its pro-oxidant character. Our results propose 3′5-DMBA as a possible candidate for IPF treatment. This is the first time to our knowledge that a maleimide has been evaluated as an antifibrotic and opens a new perspective on the use of pro-oxidants to explore their selectivity in IPF.

## Data Availability

The data that support the findings of this study are available from the corresponding author upon reasonable request.
